# Use of dupilumab to manage a grade 3 cutaneous adverse effect from enfortumab vedotin/pembrolizumab treatment in a patient with metastatic urothelial carcinoma

**DOI:** 10.1016/j.jdcr.2024.10.019

**Published:** 2024-11-09

**Authors:** Maura C. Gillis, Vanessa R. Weir, Cecilia Lezcano, Gopa Iyer, Allison Gordon

**Affiliations:** a Dermatology Service, Department of Medicine, Memorial Sloan Kettering Cancer Center, New York, New York; b Department of Pathology and Laboratory Medicine, Memorial Sloan Kettering Cancer Center, New York, New York; c Genitourinary Oncology Service, Department of Medicine, Memorial Sloan Kettering Cancer Center, New York, New York; d Department of Medicine, Weill Cornell Medical College, New York, New York

**Keywords:** bullous, dupilumab, enfortumab vedotin, flexural, oncodermatology, pembrolizumab, rash

## Introduction

While combined use of targeted cancer therapies and immune checkpoint inhibitors have successfully slowed disease progression and increased survival rates in patients with cancer, the various mechanisms of action of these anticancer drugs frequently target healthy tissues, subsequently causing dermatologic adverse events (dAEs). The nectin-4-directed antibody-drug conjugate enfortumab vedotin (EV) causes distinct dAEs such as flexural erythema, and rarely, Stevens-Johnson Syndrome/Toxic Epidermal Necrolysis (TEN).^1^ Immune checkpoint inhibitors like pembrolizumab (P) produce multiple rash phenotypes including psoriasiform, eczematous, lichenoid, vesiculobullous and hypo/hyperpigmentation.^2^ We present the case of a patient with a dAE during EV/P combination therapy for metastatic upper tract urothelial carcinoma. This case demonstrates the importance of dermatologic evaluation and diagnostic workup to elucidate the pathophysiologic mechanism driving the rash, to identify the causative drug, where possible, and to facilitate safe and appropriate treatment. Herein, we also report the successful use of dupilumab to mitigate this grade 3 dAE and successfully continue on EV/P.

## Case report

**Fig 1:**
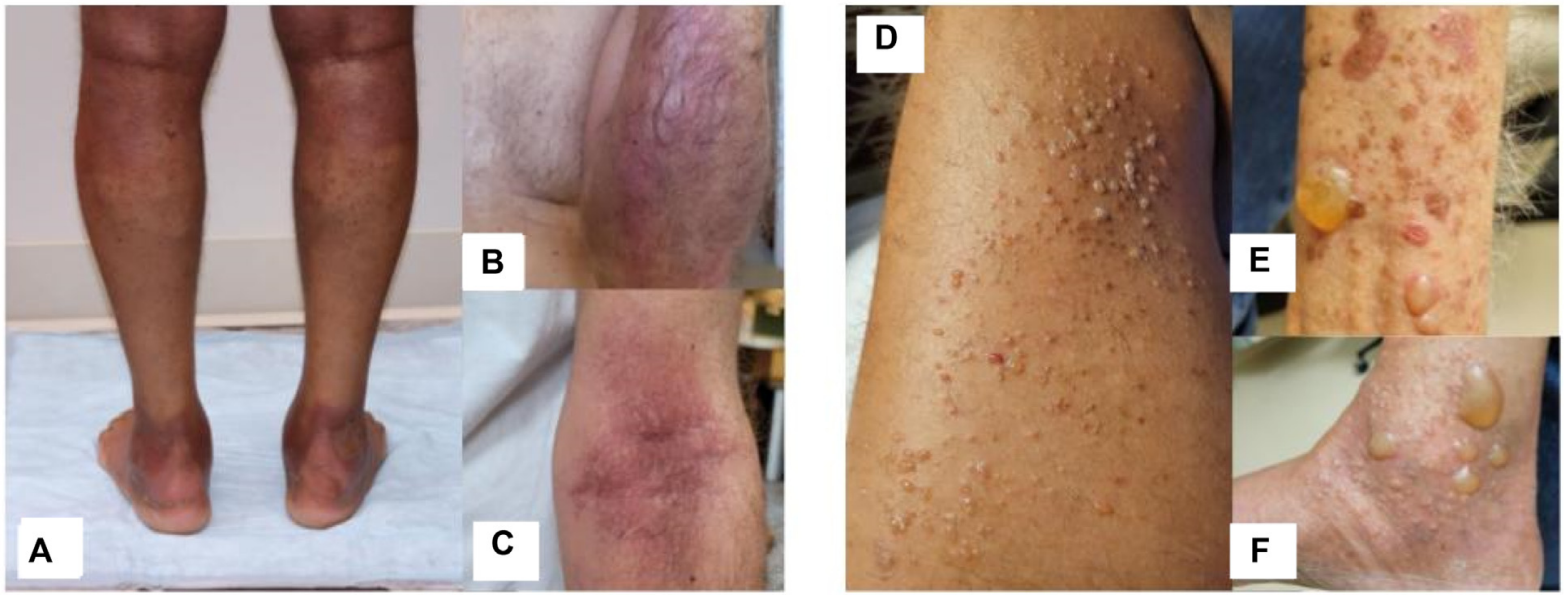
Patient presented initially with pruritic, erythematous patches on the (**A**) ankles and popliteal fossae, (**B**) posterior forearm, and (**C**) cubital fossa after the first cycle of EV/Pembrolizumab treatment. After the primary rash resolved, the patient developed a new morphology of tense, clear fluid-filled vesicles, and bullae on the (**D**) medial thighs, (**E**) ventral forearms, and (**F**) medial ankles. *EV*, Enfortumab vedotin.

A 69-year-old man with metastatic upper tract urothelial carcinoma presented to the dermatology clinic after his second cycle of EV/P with a 4-day history of pruritic, erythematous intertriginous patches without mucosal involvement (Fig 1, *A*-*C*). Initial imaging after starting EV/P showed decreased F18-fluorodeoxyglucose uptake in metastatic retroperitoneal and left supraclavicular adenopathy and no new F18-fluorodeoxyglucose avid suspicious lesions. He was prescribed betamethasone dipropionate 0.05% ointment, silver sulfadiazine cream, and pregabalin 25 mg nightly for symptomatic management of this grade 2 dAE with a recommendation to continue therapy. At 2-week follow-up and end of the third cycle, his rash had resolved with superficial desquamation. He returned to dermatology 10 days later with bilateral ankle swelling and a recurrent, progressive eruption characterized by erythematous, edematous intertriginous plaques and small, clear fluid-filled bullae that progressed despite the use of topical therapy (Fig 1, *D-F*). Mucosal surfaces were spared. The fourth cycle of EV was subsequently held, while pembrolizumab was infused. However, when his subsequent PET scan 3 weeks later revealed disease progression, the patient was rechallenged with dose-reduced EV (from 1.25 mg/kg to 1 mg/kg) given his initial robust response to this therapy. Despite continued use of superpotent topical steroids, his eruption continued to progress. The seventh cycle of EV was dose-reduced further to 0.75 mg/kg.

**Fig 2:**
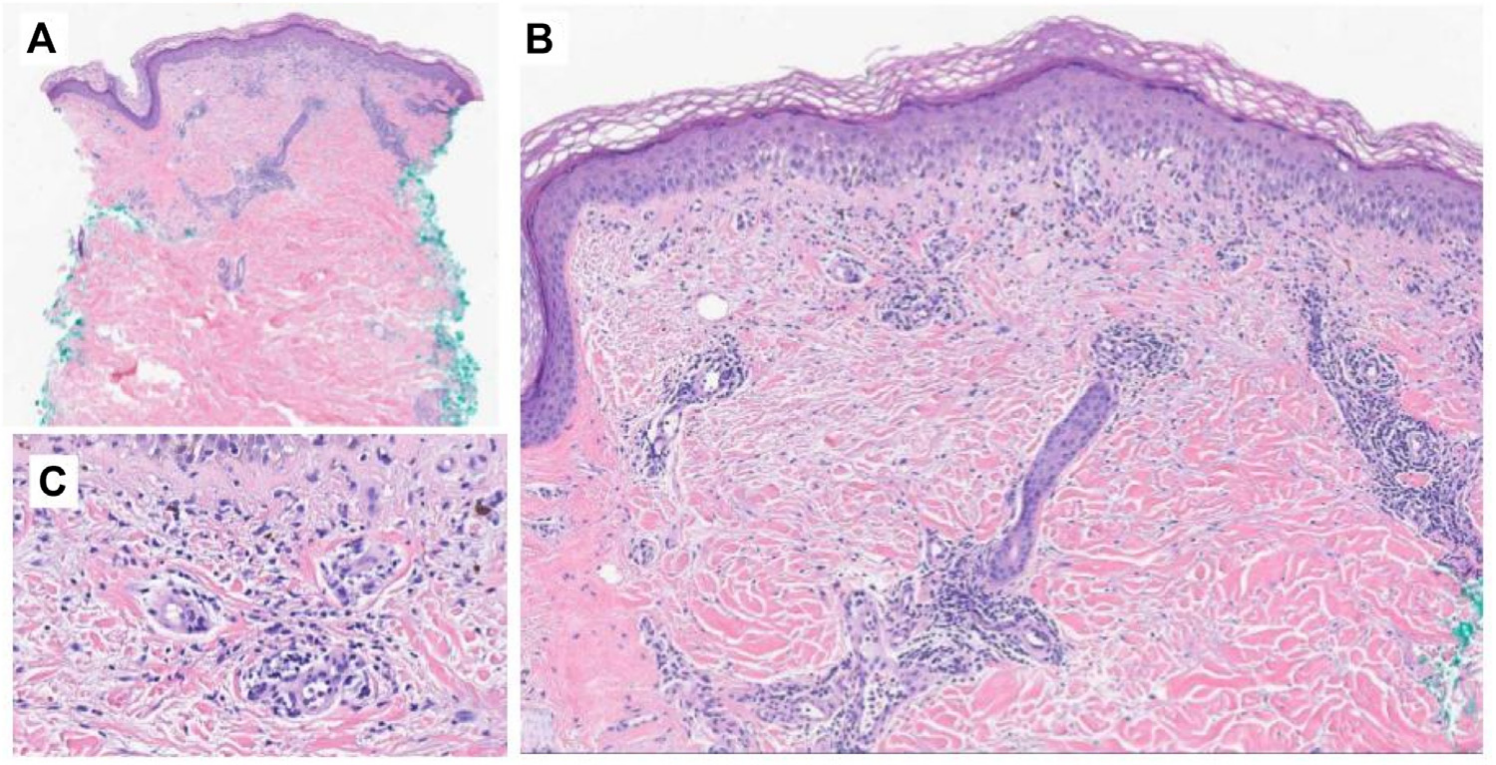
Punch biopsy of the left medial thigh showing interface dermatitis with superficial and perivascular lymphocytic infiltrate with eosinophils, suggestive of a drug reaction. **A,** Hematoxylin and eosin; magnification 2× (**B**) hematoxylin and eosin; magnification 5× (**C**) hematoxylin and eosin; magnification 20×.

**Fig 3:**
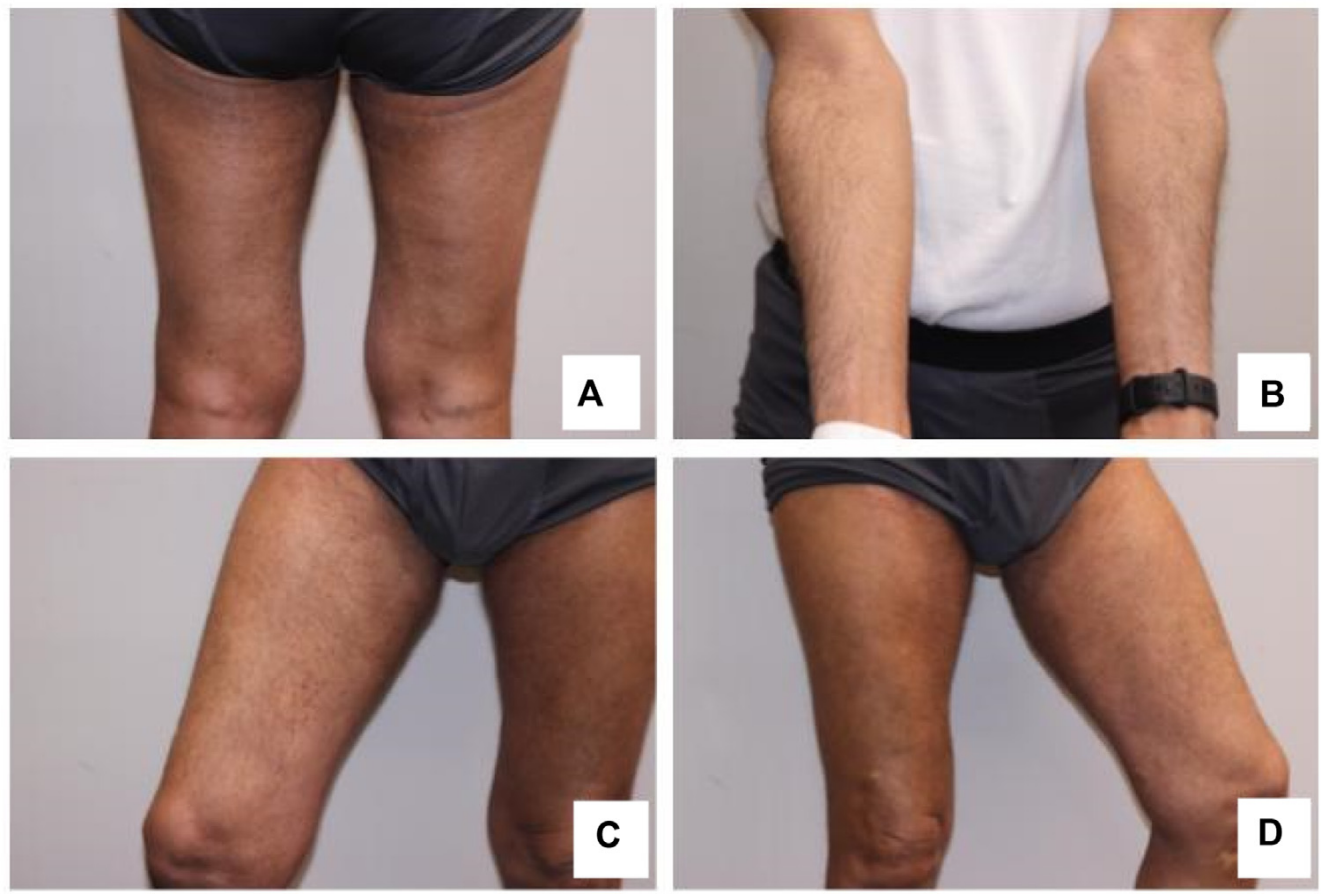
After a steroid taper, the patient was initiated on dupilumab with topical steroids and has maintained clear skin on (**A**) posterior legs, (**B**) ventral forearms, (**C**) right leg, and (**D**) left leg on reduced, but therapeutic dose of EV/P. *EV/P*, Enfortumab vedotin/pembrolizumab.

**Fig 4:**
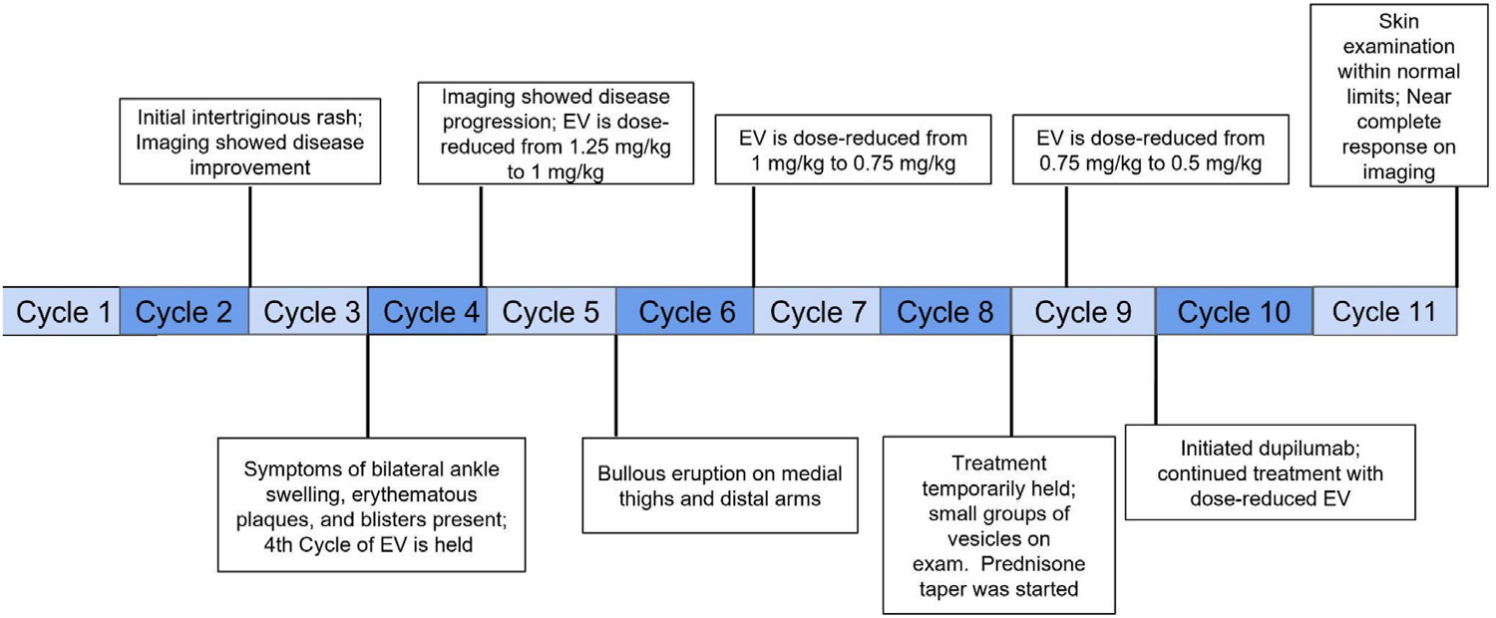
The complete timeline of EV/P treatment cycles and associated dAEs. *dAEs*, Dermatologic adverse events; *EV/P*, enfortumab vedotin/pembrolizumab.

A diagnostic workup was performed to elucidate the etiology of this eruption and help direct symptomatic therapy. This rash was a diagnostic conundrum: the pruritic vesiculobullous lesions suggested a possible PD-1 inhibitor-induced autoimmune blistering disease, whereas the intertriginous distribution suggested a direct skin toxicity-mediated rash seen commonly with EV. Two punch biopsies of representative skin of the second rash morphology were performed for hematoxylin and eosin staining and direct immunofluorescence (DIF) (Fig 2). Hematoxylin and eosin revealed interface dermatitis with superficial perivascular lymphocytic infiltrate with eosinophils; the DIF was negative. There was no syringosquamous metaplasia, keratinocyte dysmaturation, or neutrophilic infiltrate present on pathology. Serologic workup was negative for bullous pemphigoid antigen and desmoglein auto-antibodies. Taken together, the diagnostic workup suggested a bullous drug eruption but failed to show evidence of bullous pemphigoid, pemphigus vulgaris and lichen planus pemphigoides, among others. The eruption persisted to some degree despite holding and dose-reducing EV; however, we cannot definitively state that the eruption is due to pembrolizumab. A prednisone taper was initiated for immediate symptomatic control; however, given stable tumor response on sequential imaging, a long-term therapeutic option was required to maintain quiescence of skin disease while continuing EV/P. We therefore initiated off-label use of dupilumab 600 mg subcutaneous loading dose followed by 300 mg subcutaneous dosing biweekly. The patient was able to be successfully rechallenged for the 10th cycle of EV/P with EV reduced to 0.5 mg/kg and the original dose of Pembrolizumab without active skin disease (Fig 3). Additionally, imaging revealed a near-complete response to therapy after 11 cycles of EV/P. Refer to Fig 4 for a complete timeline of treatment cycles and dAEs.

## Discussion

The recently United States Food and Drug Administration-approved combination EV/P for treatment of locally advanced or metastatic urothelial carcinoma showed significantly longer progression free survival (median, 12.5 vs 6.3 months), overall survival (median, 31.5 vs 16.1 months), and a lower incidence of grade ≥3 AEs (55.9% vs 69.5%) compared to platinum-based chemotherapy.^3^ Despite an overall improved side effect profile, EV/P had more any grade dAEs, including pruritus (39.8% vs 4.8%) and maculopapular rash (32.7% vs 3.2%) than the platinum-based chemotherapy group.^3^ In patients treated with EV alone, dose reduction, interruption, and permanent discontinuation of EV due to skin toxicity was required in 8%, 11% and 4% of all patients in the treatment group, respectively.^1^ Another study suggested about 25% of patients with dAEs from checkpoint inhibitors will experience temporary or permanent discontinuation of therapy.^4^

EV is a nectin-4-directed ADC that commonly causes flexural, pruritic erythematous plaques, and rarely, dermatologic emergencies including SJS/TEN.^1^ In clinical trial, EV monotherapy demonstrated that all-grade dAEs occurred in 47% of participants.^1^ Pembrolizumab causes a variety of dAEs including pruritus, maculopapular eruptions, and autoimmune-mediated skin reactions such as depigmentation and vesiculobullous diseases such as bullous pemphigoid.^4^^,^^5^ It has been estimated that 30% to 50% of patients on immune checkpoint inhibitor therapy may experience dAEs.^4^ Our patient initially presented with an intertriginous distribution of pink plaques mimicking the classic flexural eruption seen with direct skin toxicity from EV. However, the subsequent development of tense, clear fluid-filled bullae obscured the diagnosis. A recent case series reported severe bullous flexural dermatitis in 6 patients treated with EV, with 3 patients dying within a week from multiorgan failure.^6^ Another case report presents an instance of a bullous skin eruption following EV, except there were no findings to indicate SJS.^7^

The interface dermatitis with perivascular lymphocytic infiltrate and scattered eosinophils seen on histologic analysis cannot definitively determine that a drug (and if so, which drug) caused this eruption. These histologic features are interpreted in the context of the clinical findings, time of drug administration, as well as other considerations to identify the causative agent and decide if anticancer therapy can be safely continued with appropriate measures. The IL-4 receptor antagonist dupilumab is United States Food and Drug Administration-approved for atopic dermatitis and prurigo nodularis, among other non-dermatologic diagnoses, and has anecdotally shown efficacy for steroid-refractory immune checkpoint inhibitor-induced spongiotic and interface dermatitis.^8^^,^^9^ Herein, we demonstrate the successful off-label use of dupilumab to manage an EV/P-related dAE of mixed morphology. The mitigation of our patient’s rash permitted him to remain on his anticancer treatment course to date, nearly 12 weeks after initiating dupilumab without any active dermatologic disease and a near-complete response to therapy. This report highlights the importance of collaboration between supportive oncodermatologists and oncologists to diagnose dAEs, employ diagnostic tests to identify culprit drugs, and therapeutically manage such adverse effects so that patients can remain on life-prolonging therapies. Finally, we show the utility of dupilumab as a successful therapy in managing severe dermatologic toxicity caused by the novel EV/P combination treatment.

## Conflicts of interest

Dr Iyer receives funding from Flare Therapeutics, Loxo at Lilly, Pfizer, and Aadi Biosciences. Dr Iyer also receives consulting fees from Pfizer, Loxo at Lilly, and Aadi Biosciences. Drs Gordon and Lezcano and Authors Gillis and Weir have no conflicts of interest to declare.
